# Typology of content warnings and trigger warnings: Systematic review

**DOI:** 10.1371/journal.pone.0266722

**Published:** 2022-05-04

**Authors:** Ashleigh Charles, Laurie Hare-Duke, Hannah Nudds, Donna Franklin, Joy Llewellyn-Beardsley, Stefan Rennick-Egglestone, Onni Gust, Fiona Ng, Elizabeth Evans, Emily Knox, Ellen Townsend, Caroline Yeo, Mike Slade

**Affiliations:** 1 School of Health Sciences, Institute of Mental Health, University of Nottingham, Nottingham, United Kingdom; 2 Narrative Experience Online Lived Experience Advisory Panel, Nottingham, United Kingdom; 3 Department of History, University of Nottingham, Nottingham, United Kingdom; 4 School of Cultures, Languages, and Area Studies, University of Nottingham, Nottingham, United Kingdom; 5 School of Information Sciences, University of Illinois at Urbana-Champaign, Champaign, Illinois, United States of America; 6 School of Psychology, University of Nottingham, Nottingham, United Kingdom; University of Michigan, UNITED STATES

## Abstract

Content and trigger warnings give information about the content of material prior to receiving it. Different typologies of content warnings have emerged across multiple sectors, including health, social media, education and entertainment. Benefits arising from their use are contested, with recent empirical evidence from educational sectors suggesting they may raise anxiety and reinforce the centrality of trauma experience to identity, whilst benefits relate to increased individual agency in making informed decisions about engaging with content. Research is hampered by the absence of a shared inter-sectoral typology of warnings. The aims of this systematic review are to develop a typology of content warnings and to identify the contexts in which content warnings are used. The review was pre-registered (ID: CRD42020197687, URL: https://www.crd.york.ac.uk/prospero/display_record.php?ID=CRD42020197687) and used five sources: electronic databases covering multiple sectors (n = 19); table of contents from multi-sectoral journals (n = 5), traditional and social media websites (n = 53 spanning 36 countries); forward and backward citation tracking; and expert consultation (n = 15). In total, 6,254 documents were reviewed for eligibility and 136 documents from 32 countries were included. These were synthesised to develop the Narrative Experiences Online (NEON) content warning typology, which comprises 14 domains: Violence, Sex, Stigma, Disturbing content, Language, Risky behaviours, Mental health, Death, Parental guidance, Crime, Abuse, Socio-political, Flashing lights and Objects. Ten sectors were identified: Education, Audio-visual industries, Games and Apps, Media studies, Social sciences, Comic books, Social media, Music, Mental health, and Science and Technology. Presentation formats (n = 15) comprised: education materials, film, games, websites, television, books, social media, verbally, print media, apps, radio, music, research, DVD/video and policy document. The NEON content warning typology provides a framework for consistent warning use and specification of key contextual information (sector, presentation format, target audience) in future content warning research, allowing personalisation of content warnings and investigation of global sociopolitical trends over time.

## Introduction

Trigger warnings and content warnings are “*a statement at the start of a piece of writing*, *video*, *etc*. *alerting the reader or viewer to the fact that it contains potentially distressing material–often used to introduce a description of such content*” (p.602) [1]. This dual use–both warning about, and characterising the content of, material–may account for the terms ‘trigger warning’ and ‘content warning’ being used interchangeably across the diverse academic disciplines which research their use. Some but not all studies locate trigger warnings as a particular sub-type of content warnings which are focussed specifically on the needs of people with experience of trauma or post-traumatic stress disorder (PTSD). In addition, the use of ‘trigger’ has been disputed because what constitutes a ‘trigger’ in relationship to PTSD is highly individual and unpredictable [2]. In this paper we therefore use the general term ‘content warning’.

The use of content warnings is longstanding and widespread across multiple sectors, e.g. health, education, media, arts and literature. The concept of ‘triggering’ describes the re-experiencing of unpleasant PTSD symptoms such as intrusive thoughts being evoked by exposure to materials which spark traumatic memories. Hence, content warnings have a long presence in psychiatric literature [3]. Similarly, most countries use classification systems for film and television media, such as Australia [4] and Singapore [5]. For example, a film rating classification system has been used in the United Kingdom (UK) since 1912. In education, use of content warnings is widespread [6], e.g. a 2016 survey of professors in the United States of America (USA) found that 428 (51%) of the 841 respondents reported some use of content warnings in their classes [7]. Content warnings have also been advocated and used in other sectors, including books [8], comics [9] and museums [10]. Multinational frameworks have been established, such as the Pan European Game Information (PEGI) categories of age labels [3, 7, 12, 16, 18] and content descriptors (Bad language, Discrimination, Drugs, Fear, Gambling, Sex, Violence, In-game purchases) for computer games.

There is no inter-sectoral consensus or widely used typology of content warnings. Incompatible frameworks have been developed, driven by the perceived need for different uses and in different legal and geographical jurisdictions. One reason for differences in content warning is the un-coordinated development of frameworks across different sectors. Each sector contains different assumptions, so for example in the arts sector provocation caused by displayed materials is valued and intended [11], with content warnings primarily giving information so individuals can make their own choices about exposure. By contrast, in the health sector the biomedical ethical imperative of non-maleficence [12] places more responsibility on the health professional to actively reduce the likelihood of triggering trauma responses in people with experience of PTSD. Hence, the content warnings in arts spaces tend to be more focussed on information, whereas those in a health context contain less information to avoid the content warning itself being triggering.

The development of shared inter-sectoral practices such as consensus on a content warning typology is made more difficult because of the contested evidence base. Broadly, some researchers argue that content warnings are a form of over-protection which inadvertently hinder the development of resilience [13], whereas others argue that content warning allows both avoidance of un-wanted exposure experiences and emotional preparation to reduce negative reaction to content [14]. More recent studies have experimentally investigated the impact of content warnings, especially in educational settings. A randomised study found participants with no trauma history (n = 133) who received warnings before reading passages with disturbing content reported more anxiety than those not receiving (n = 137) warnings, suggesting warnings can undermine emotional resilience [15]. The same authors replicated this finding with a college student sample (n = 462) [16], and also showed in a randomised study of trauma survivors (n = 451) that content warnings inadvertently reinforce the centrality of trauma experiences to identity [17]. A 2019 meta-analysis confirmed this finding that content warnings are associated with increased anxiety and negative mood [1]. Meta-analyses of a series of studies involving students and internet volunteers, with and without a trauma history, found mainly neutral or slightly negative impact of content warnings, leading the authors to conclude that such warnings are neither meaningfully helpful or harmful [18].

Given the emerging empirical evidence base, and the publication of balanced and authoritative overviews [19], why is there difficulty in reaching consensus? Despite early suggestions in the 2000s of a new interdisciplinary area emerging called warning research [20], and signs of social media conventions developing [21, 22], much of the public discussion about content warnings has been heated. The use of emotive language such as the ‘trigger warning war’ [23] is perhaps related to wider cultural debates and potentially clashing priorities, for example free speech and censorship approaches versus trauma-informed and rights-informed approaches. The issue is international, and passionate articles have been written by authors in Australia [24], Ireland [25], the UK [26] and the USA [27]. For example, some have argued that, whatever the experimental evidence, content warnings are a vital approach to increasing inclusivity on campuses because they can support engagement by people who would otherwise avoid material due to past experiences [28]. Others cite evidence that content warnings can be useful in specific educational contexts, such as victimology courses [29].

An inter-sectoral typology of content warnings would advance the field, in three ways. First, by allowing the comparability of findings from across different studies to be maximised. At present there is no recommended typology, and so empirical studies use different content warnings for the same content. Identifying an agreed description for a warning and locating it in a broader and coherent typology will support more fine-grained investigation about the impact of specific types of warning. In addition, the typology will be of interest to a range of fields, including arts, media studies, medicine, mental health, and psychology. Second, the limits of content warnings would be helpful to establish, to understand what is in scope. For example, are ‘Contains nuts’, ‘Contains flashing imagery’ and the perhaps ironic ‘Depicts killer robots’ all appropriately understood as content warnings? Finally, identifying the typology, sector, presentation format and target population will allow more specific future research into the positive and negative impacts of content warning when used in a specific sector with a specific target population.

The aims of this review are (1) to develop a typology of content warnings, and (2) to identify the contexts in which content warnings are used, comprising the sector (e.g. education, health), format (e.g. film, music) and target audience.

## Method

The protocol of this systematic review was developed in accordance with PRISMA guidelines and was registered on PROSPERO (International Prospective Register of Systematic Reviews) on 9 July 2020 (reference CRD42020197687). The study was conducted as part of the Narrative Experiences Online (NEON) study (http://www.researchintorecovery.com/neon), which aims to evaluate the impact of recorded recovery narratives [30] when used as a mental health intervention [31]. In developing the intervention [32], we needed to decide whether to use content warnings when delivering recorded recovery narratives to participants in randomised controlled trials for people with experience of psychosis (NEON Trial: ISRCTN11152837), for people with experience of non-psychosis mental health problems (NEON-O Trial: ISRCTN63197153), and for informal mental health carers (NEON-C trial: ISRCTN76355273) [33]. The typology developed in this review is therefore called the NEON content warning typology.

### Eligibility criteria

Studies were included where the document (a) reported a text-based list, set or typology of content warnings, or (b) presented empirical evidence or a structured framework regarding the context (e.g. target audience, sector, and presentation format) for the use of specific content warning. We included both peer-reviewed and non-peer reviewed literature, including empirical studies of any design (e.g. surveys, experiments, qualitative interviews), commentaries, opinion pieces, media organisation web-pages, and systematic and non-systematic literature reviews. No restrictions were placed on the population of study. We excluded studies that were non-English language documents and published before the year 2000 as a high-quality review was published in 2002 [34].

### Information sources

Five data sources were used:

1. Electronic bibliographic databases (n = 19) from a range of academic disciplines and sectors were searched: ACM Digital Library, Applied Social Sciences Index and Abstracts (ASSIA), CINAHL, Education Database, Education Resources and Information Center (ERIC), e-theses online service (EThOS), IEEE Xplore, International Bibliography of the Social Sciences (IBSS), JSTOR, Library & Information Science Source, MEDLINE, Project MUSE, ProQuest Dissertation and Theses Global, PsycINFO, PubMed, Sociological Abstracts, Social Science Database, Sociology Database, and OpenGrey.
2. The table of contents from journals (n = 5) spanning multiple sectors as selected by topic experts: Communication Education; Feminist Teacher; Game Studies; Participations: Journal of Audience & Reception Studies; and Suicide and Life-Threatening Behaviour.
3. Traditional and social media organisation websites (n = 53) relating to 36 countries (Australia, Austria, Belgium, Brazil, Bulgaria, Canada, Denmark, Finland, France, Germany, Hong Kong, Iceland, India, Indonesia, Ireland, Jamaica, Japan, Malaysia, Maldives, Netherlands, New Zealand, Nigeria, Norway, Malta, Philippines, Poland, Saudi Arabia, Singapore, South Africa, South Korea, Sweden, Taiwan, Turkey, United Arab Emirates, United Kingdom and United States of America) and 12 international organisations (Amazon, Apple Store, Blackberry, Entertainment Software Rating Board (ESRB), Facebook, Google Play, International Age Rating Coalition (IARC), Netflix, Pan European Game Information (PEGI), Reddit, Twitter, YouTube). These were identified through online searching to find English-language classification guidance and are listed in S1 Appendix.
4. Forward citation tracking was performed on all included studies using Google Scholar and backward citation tracking by hand-searching reference lists of all included studies.
5. Consultation with the multidisciplinary authorship team (n = 11) and other international experts (n = 4) was used to identify additional key documents or studies.

### Search strategy

Through a combination of a preliminary scoping search and expert consultation we identified five sectors which we aimed to cover in our search strategy: education (primary, secondary and tertiary), healthcare (medicine, and mental health/psychology), media (television, film, social media and gaming), human rights (feminist and gender studies, critical race studies), and digital technologies (computing and information sciences). Search terms used were ‘advisory warning’, ‘content note’, ‘content notice’, ‘content warning’, ‘trauma trigger’, ‘trigger warning’, and ‘video nasty’. The search terms were modified for each database, for example the search strategy used for PsycINFO and MEDLINE was: ("content warning" OR "trigger warning" OR "content notice" OR "content note" OR "advisory warning" OR "trauma trigger" OR "video nast*").ti,ab. All database searches were conducted from the year 2000 to the search date.

### Study selection

For all searches, identified citations were collated and uploaded to EndNote X9. After removing duplicates, the titles and abstracts of all identified citations were screened for relevance against the inclusion criteria by three analysts (AC, HN and LHD) with a randomly-selected subsample (5%) independently assessed by LHD. Concordance between reviewers was 100%. Full texts were screened by AC, HN and LHD with a randomly-selected subsample independently assessed by LHD (10%), showing 100% concordance.

### Data abstraction

For each document, information was extracted on:

1. Document characteristics, comprising publication year, peer reviewed journal, publication type, sector, and country of affiliation of the lead/first author
2. Content/trigger warning list characteristics, comprising function of list (listing categories vs. discussing warning use in specific contexts), type of warning list (‘trigger warning’ vs. ‘content warning’ or synonyms), warning labels (the list of warnings), source reference, presentation format (e.g. ‘classroom’, ‘film’), target audience, number of lists in the document
3. Exclusion/inclusion criteria, sample size, and study design [empirical studies only]
4. Country and setting of list use.

The data abstraction table (DAT) is shown in S2 Appendix. The DAT was piloted by AC and LHD who independently abstracted a randomly selected 10% of the included documents. Concordance was 92%. The DAT was then refined following discussion between reviewers and agreement was reached on developing instructions for further abstraction. AC, DF, HN and LHD then independently extracted data from the remaining documents.

### Quality assessment

The quality of the included documents which reported an empirical study were assessed using the Mixed Methods Appraisal Tool (MMAT) [35]. The MMAT has sections for different types of study, each with its own set of methodological quality criteria: (1) qualitative; (2) quantitative randomised controlled trials; (3) quantitative non-randomized; (4) quantitative descriptive; and (5) mixed methods. For each item the answer categories were ‘Yes’, ‘No’, or ‘Can’t tell’ followed by comments. The MMAT was chosen as it covers the range of empirical studies involved in this review and has moderate-to-excellent inter-rater reliability. No quality assessment was made of those documents not reporting empirical research as there are no standard criteria or processes for assessing the quality of such documents.

### Data synthesis

Data synthesis was conducted on included papers. The two primary analysts (AC, MS) came from different mental health professional (nursing and clinical psychology) and academic (sociology and health research) backgrounds, and the other analysts (LHD, HN, DF, JLB, SRE, OG, FN, EE, EK, ET, CY) came from varied disciplinary and sectoral backgrounds (heath service research, psychology, sociology, youth work, social science, history, media studies).

For objective 1 (developing a typology of content warnings), warning lists and items from all the included studies were synthesised using the following process: a) duplicates of content warning items were removed; b) content warnings which perpetuate structural inequality were removed (the only instance found was a warning about same-sex marriage, parenting or sexual activity found in six included documents); c) labels (codes) were developed for important features in the data; d) initial categories were created by examining the codes and identifying significant broader patterns of meaning; e) categories were reviewed through checking the candidate categories against the dataset, in order to determine whether they closely mapped onto the data. Once confirmed, the wording of the label was reviewed to ensure maximum comprehensibility, e.g. anti-disability was chosen over ableism; and f) vote counting of number of papers identifying each category was performed to establish the strength of each category. The preliminary analysis was conducted by AC and MS. The analysis was then iteratively refined in discussion with all co-authors.

## Results

**Fig 1:**
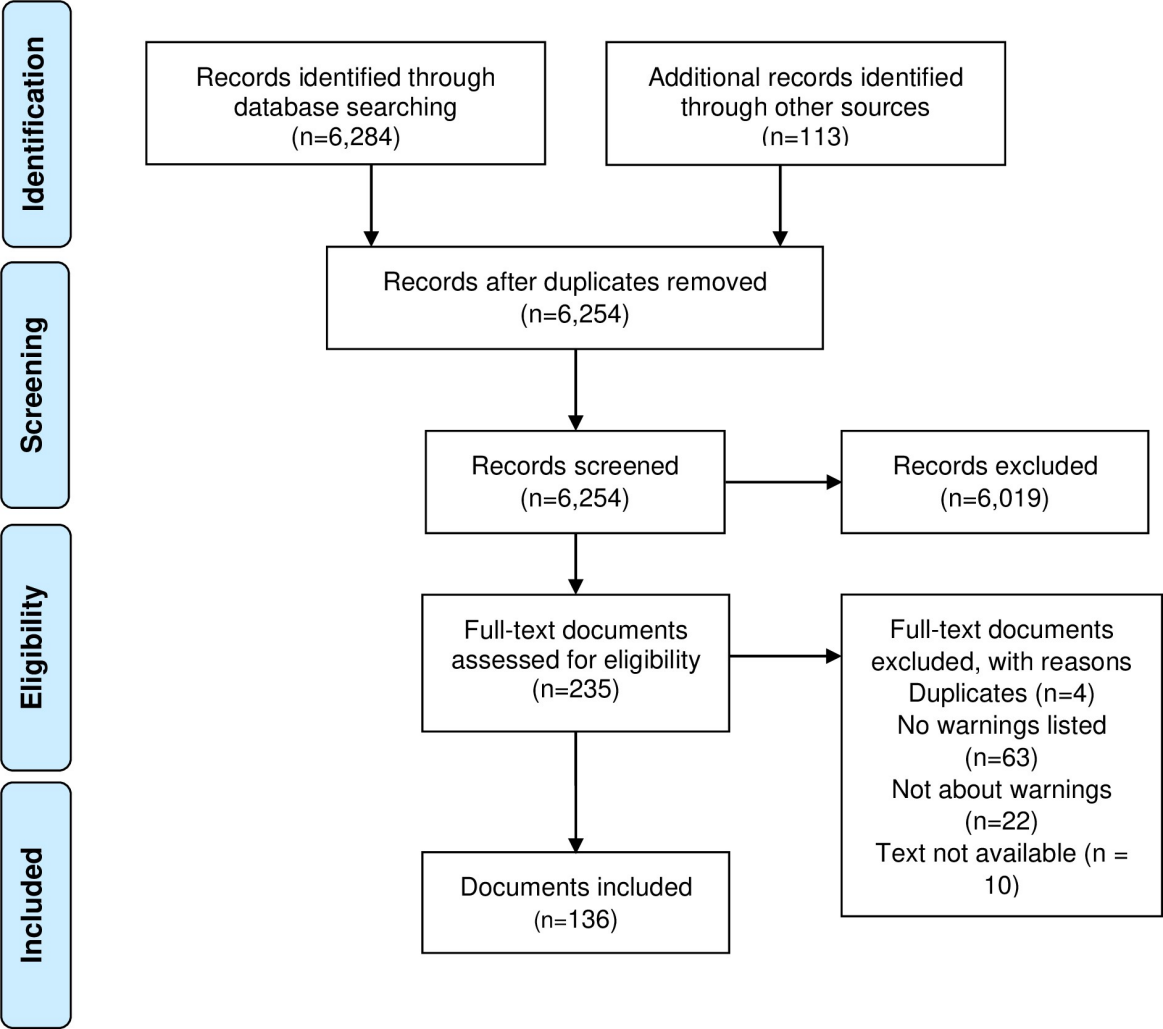
Flow diagram for included documents.

The search identified 6,254 documents, from which 136 were included. The flow diagram is shown in Fig 1.

The data abstraction table for all included documents is shown in S2 Appendix. The 136 included documents comprised webpages (n = 43), academic journals (n = 38), newspaper/magazines (n = 36), technical reports (n = 12), books (n = 4), conference abstracts (n = 2), and a thesis (n = 1). Of these, 38 (28%) documents were peer-reviewed studies and 98 (72%) were non-peer reviewed. The included documents came from 32 countries covering North America (n = 74), Europe (n = 22), international which covered multiple countries (n = 15), Asia (n = 14), Australasia (n = 4), Africa (n = 3), both Europe and USA (n = 1), and South America (n = 1). The majority of documents came from the USA (n = 69) (51%).

Only 18 (10%) documents reported empirical studies, comprising observational (n = 9), experimental (n = 6), qualitative (n = 2), and mixed methods (n = 1) designs. As a result of this small proportion, it was not appropriate to conduct a sensitivity analysis comparing higher to lower quality studies. Therefore, the MMAT ratings, although retained in the data abstraction table, were not used in the analysis.

Across the 136 documents, 330 warning lists were identified containing a total of 2,209 warnings (including duplicates). The documents comprised 52 (16%) defining a list of warnings and 278 (84%) outlining specific warnings for use in a specific context, e.g. classroom. Across all lists, 134 (41%) were labelled as trigger warnings and 196 (59%) as content warnings.

### Objective 1: NEON content warning typology

The 2,209 warnings were synthesised into 14 categories of content warning, as shown in Table 1.

**Table 1:** NEON content warning typology.

Category (n) and definition	Sub-categories
**1. Violence** (n = 536)	Violence; War; Weapons; Terrorism; Police brutality; Motiveless killing; Sexual violence; Animal cruelty; Torture; Genocide
Content contains violence	
**2. Sex** (n = 332)	Nudity; Mild sexual content; Explicit sexual content; Relationship conflict; Reproductive health
Content contains sexual themes, including nudity, sexual content and relationships	
**3. Stigma** (n = 328)	Racism; Anti-religious (sub-categories: Anti-Semitic; Anti-Christian; Islamophobia); Colonialism; (sub-category: Slavery); Classism; Sexism (sub-categories: Misogyny; Misandry); Transphobia; Gender-identity; Sexuality (sub-category: Homophobia); Anti-disability
Content depicts negative stereotypes about or attitudes towards a specific group, such as racism or sexism	
**4. Disturbing Content** (n = 236)	Disturbing content with threat; Horror and terror; Disturbing imagery; Medical content; Human bodies and functions
Content contains imagery, sounds, or effects that may frighten, disgust or scare	
**5. Language** (n = 235)	Sexual language; Adult humour; Swearing; Offensive language
Content contains language which is sexual, crude or offensive	
**6. Risky Behaviours** (n = 200)	Drug misuse; Alcohol misuse; Tobacco; Gambling
Content depicts risky lifestyle behaviours	
**7. Mental Health** (n = 108)	Mental health; Eating disorders; Trauma; Self-harm and suicide; Depression; OCD; Panic attacks; Anxiety (sub- categories: Spiders; Snakes; Insects; Needles; Eye contact; Irregular patterns); Hair pulling
Content relates to mental health issues	
**8. Death** (n = 49)	Death; Accidents; Natural disasters
Content relates to human death or dying	
**9. Parental Guidance** (n = 47)	Online access; Cyber-bullying; Competitive content; Imitative content; Upsetting content; Non-realistic violence
Content may not be appropriate for children	
**10. Crime** (n = 38)	
Content depicts or relates to criminal activity	
**11. Abuse** (n = 37)	Child abuse; Emotional abuse; Physical/sexual abuse; Neglect
Content depicts or relates to abuse	
**12. Sociopolitical** (n = 27)	Injustice; Political issues; Nazism; Class issues
Content includes social or political issues	
**13. Flashing Lights** (n = 27)	
Content includes strobe or flashing lighting	
**14. Objects** (n = 4)	
Content contains specific objects	

The complete NEON content warning typology, including definition, number of documents using the category, and example text for all sub-categories, is shown in S3 Appendix.

## Objective 2: Contexts for content warning use

Ten sectors using content warnings were identified in the 136 documents: education (n = 53), audio-visual industries (n = 34), games and apps (n = 18), media studies (n = 4), social sciences (n = 3), comic books (n = 3), social media (n = 3), music (n = 2), mental health (n = 2), science and technology (n = 1). Thirteen documents spanned multiple sectors. Compared to other sectors, Education had the most frequent mentions of warnings relating to Violence, Sex, Stigma, Risky behaviour, Mental health, Abuse, Crime, Socio-political, and Objects. Audio-visual industries were the highest users of warnings about Disturbing content, Language, Death, and Parental guidance. Games and apps were the sector most using warnings about Flashing lights.

A total of 15 presentation contexts for the use of content warnings were identified in the 330 lists: education materials (n = 95), film (n = 48), games (n = 43), websites (n = 32), television (n = 16), books (n = 15), social media (n = 9), verbally (n = 5), print media (n = 4), apps (n = 3), radio (n = 2), music (n = 2), research (n = 1), DVD/video (n = 1), policy documents (n = 1) and multiple contexts (e.g. classroom and online forums) (n = 59). Educational materials was the most frequently used presentation format for content warnings relating to Violence, Sex, Stigma, Mental health, Abuse, Socio-political, and Objects. Disturbing content, Language, and Risky behaviours content warning categories were most frequently used in Games. Film was the most used presentation format for Death, Parental guidance and Crime.

The target audience in the 330 warning lists comprised mainly the general public (n = 197) and students (n = 119), with other recipient groups identified including children and adolescents (n = 8), women (n = 3), parents (n = 2), and people with post-traumatic stress disorder (PTSD) (n = 1). Generally, the included papers did not explicitly state the target audience for the content warning, so the target audience was recorded as general public. Students were a highly cited target audience across all warning categories, and were more cited than the general public for the Mental health warning. For the remaining thirteen warning categories, the most cited target audience was the general public.

## Discussion

This systematic review included documents from 32 countries and developed the NEON content warning typology comprising 14 categories of warning. The categories of Violence, Sex and Stigma were the most widely used warnings, but some warnings were more sector-specific, such as Parental Guidance in relation to films. Ten distinct sectors in which content warnings are used were identified, along with 15 distinct presentation formats. The target audience for content warning usage was often not explicitly stated.

Several principles informed the typology development. First, content warnings relating to media content and not product safety were included. For example, product labelling for food (e.g. Contains nuts) and cleaning products (e.g. Keep out of reach of children) were deemed out of scope in this review. The category of Flashing Lights was a boundary condition which was included due to its use in films, even though it is also used on consumer products such as toys.

Second, the need for accessible language informed label choices where possible, e.g. Anti-disability was chosen over Ableism. However, preference was given to retaining the meaning, e.g. Misogyny and Misandry were selected since labels such as Anti-female and Anti-male did not adequately capture the full meaning of gender-based prejudice. These terms are spreading due to increased use and the increasing rights literacy among younger people [36].

Finally, the NEON content warning typology is designed to be extendable in scope and depth. This may be needed for several reasons.

- The decision to exclude warnings that perpetuate structural inequality as part of the synthesis led to the exclusion of a Same-sex relationship warning, in order to avoid replicating heteronormative assumptions and prejudice. Alternative or additional approaches to preventing the perpetuation of structural inequality could be considered.
- The categories in the typology are not wholly distinct. For example, Anti-Semitic was positioned in the typology as a sub-category of Anti-religious due to its use in included documents, but could also be located as a form of Racism. Similarly, there is an inter-linkage between the Stigma and Sociopolitical categories, and the challenges of locating categories in either (e.g. Racism and Sexism in Stigma and Injustice in Sociopolitical) or both (e.g. Classism in Stigma and Class issues in Sociopolitical) reflect that these complex issues can be viewed both as individual and structural problems. Similarly, warnings about depictions of violence in relationships may draw from both the Violence and Abuse categories.
- The typology was constrained to reflect the content of included documents. This meant that some categories would benefit from future disaggregation or elaboration. For example, Political issues incorporates both pro-capitalist and anti-capitalist content, so a future iteration may develop sub-categories to differentiate these two very different concerns. The Risky Behaviours category currently does not include any online behaviours, which may be a future extension. Other candidate extensions include a Pro-religious warning in the Stigma category and a Sexualising of children warning in the Abuse category.

Globally, the translation of content warnings is an important consideration. For example, content warnings in media and printed materials may vary between differing countries and resource settings due to specific cultural and contextual factors, mediated by the power held by organisations that specify the use of content warnings, such as film classification bodies. Examples might include different thresholds in relation to depictions of sexual behaviour, drug use and clothing, which can change over time. In addition, content warnings may not be used in some settings, highlighting the prominence of content warnings in particular contexts such as higher education. The translation and use of content warnings in other settings will need to be considered. Methodologies now exist to support the proportionate translation of the content warnings into other languages [37]. In relation to use, when presenting stories in different settings, as will be done in the NEON study, specific cultural or contextual content warnings may need to be included to meet the needs of recipients from different cultural backgrounds.

Overall, the typology is designed to be extended, as social norms evolve and as research into content warnings develops. The NEON content warning typology reflects current inter-sectoral research and practice, and so refinement within its overall structure is actively encouraged and recommended.

### Strengths and limitations

Several strengths can be identified. This is the first systematic review proposing an inter-sectoral typology. Previous systematic reviews have focused on individual warnings for specific products, such as cigarettes [38] or alcohol [39]. A second strength of the NEON content warning typology is that it is based on current practice, inter-sectoral research and literature, not within-sector expertise. For example, the Mental health sub-category of Irregular patterns was included which is a rare problem from a psychiatric epidemiological perspective, whereas diagnoses such as Claustrophobia are more prevalent. Starting from a mental health sectoral perspective could lead to simply reproducing sector-specific taxonomies as a content warning typology, which would limit inter-sectoral applicability. Further strengths include the wide range of sources from multiple sectors and countries, and the cross-disciplinary multi-analyst approach used in data synthesis.

Limitations include the findings that the majority (51%) of included documents came from the USA, reflecting the strong focus on content warnings in that country but raising the question of generalisability of findings. The extendibility of the NEON content warning typology is an important feature to address potential ethnocentrism. A second limitation is the inability to use the study quality metrics, due to the small proportion of scientific studies in the included documents. A third limitation relates to the inclusion criteria requiring papers to use text-based warnings, which excluded other types of warning such as graphic icon images. Future work might develop icons for each of the 14 categories in the typology.

## Implications

The study has three implications for future research. First, this review can support the emergence of a coherent and aggregable evidence base allowing the impact of content warnings to be more systematically investigated. The use of content warnings relates to other safety-driven initiatives. A recent systematic review investigating removal or blurring of self-harm online imagery found parallel issues of potential harm and positive impacts [40], reinforcing that research in content warnings and cognate areas is complex. There is a need to move beyond the current somewhat simplistic focus on whether content warnings help or harm towards a contextualised understanding of the mechanisms by which content warnings impact on recipients with different characteristics in different sectors and when used for different purposes. Future content warning studies should clearly describe the specific warnings used and the context including sector, presentation format and target audience. Using the NEON content warning typology and identified usage descriptors such as the names for the ten sectors will increase opportunities to integrate the currently diverse evidence base. A recommended citation terminology for use in studies using the NEON content warning typology would be identifying the specific warning(s) using the category number and name (e.g. ‘NEON 1.3 Weapons’) and specifying the sector, format and target audience involved in the research. This review found no consensus about the use of the term ‘trigger warning’ versus ‘content warning’, so to ensure relevant studies can be easily located in future reviews it is recommended that abstracts for relevant studies always include the term ‘content warning’. Overall, these recommendations will support the development of a more robust and integrated scientific evidence base, which can illuminate issues such as the optimal granularity of a warning (Violence? Motiveless killing?) and the mechanisms of impact on different groups in different contexts.

A second and related future focus could be on a more fine-grained perspective about differential impacts of content warnings on specific target audiences. Most included documents did not explicitly specify the audience, which is an important omission. For example, there is emerging evidence that post-traumatic growth in psychosis is more common than expected [41, 42], which may inform the use or not of content warnings with this clinical population. Similarly, greater clarity about the relationship between positive and negative impacts of warnings on specific audiences would allow personalisation of individually delivered content warnings in online apps, games, and web-browsing. For example, web-browser extensions have been developed which automatically detect potentially sensitive content warnings for specific topics, with the goal of creating safe internet spaces for users [43]. Whilst The NEON content warning typology presents a list of content warnings, it is limited to reflect the included documents. However, the typology could inform creators of such technologies about what content warnings exist and what topics and/or words need to be included and screened for. Similarly, for audiences, future web-based innovations may allow individuals to choose what specific content warnings to screen that meets their needs, and the typology can support individuals in deciding which specific topics/words to include or exclude. Further personalisation of content may also include recording recipient characteristics as part of a personal profile on a smartphone which would allow apps to be tailored to include content warnings tailored to individuals with particular characteristics, or to include content warnings only for some recipients based on their personal profile.

A third area of research is to investigate secular or time trends in the use of different types of warning. For example, in this study Stigma was one of the most widely used categories, which may reflect an increasing global focus on issues of rights and discrimination, and the links between being a member of a stigmatised group and experiencing trauma [44]. Future research might investigate whether the changing pattern of content warning use over time can be used as a barometer to capture global socio-political trends, such as the increasing recognition of the ongoing trauma effected by historical colonialism, racism, and trans-Atlantic slavery. Changing patterns of content warning use may in this way illuminate wider societal changes, giving a new source of data for social science research seeking to characterise the evolution of societal values and priorities.

Finally, the next stage in research may involve addressing the short-term nature of content warnings. The NEON content warning typology provides a framework for alerting to potential distressing and/or sensitive content, but in other environments such as academia, content warnings are provided alongside faculty and student service support. Evaluation will need to identify if any additional resources beyond the typology alone are needed to support recipients when content warnings are in different settings, and whether the typology is useful for recipients in deciding what content to engage with.

Overall, the NEON content warning typology is an empirically-defensible theoretical foundation for future content warnings research. It has been developed by analysts from diverse professional and academic perspectives, enhancing its intersectoral applicability. Using the typology will support investigation addressing the important and currently under-researched question of how different types of content warnings impact on different audiences in different sectors.

## Supporting information

S1 Checklist PRISMA 2020 checklist. (DOCX)

S1 Appendix Media. (DOCX)

S2 Appendix Data abstraction table. (XLSX)

S3 Appendix Complete coding framework. (DOCX)
