# Supplementary material for: Typology of content warnings and trigger warnings: Systematic review
Source: PLoS One. 2022 May 4;17(5):e0266722. doi: 10.1371/journal.pone.0266722 (PMC9067675; doi:10.1371/journal.pone.0266722)
Supplement: S1 Appendix — (DOCX) [file pone.0266722.s002.docx]

**Appendix S1**

**National and international media organisations reviewed (n=53)**

| **Country** | **Organisation(s)** | **Web-site(s) reviewed** |
| --- | --- | --- |
| Australia | Australian Classification Board (ACB) | https://www.classification.gov.au/classification-ratings/what-do-ratings-mean |
| Austria | Austrian Board of Media Classification (ABMC) | https://www.kijkwijzer.nl/kijkwijzer |
| Belgium | Adopted the Dutch Kijkwijzer classification system | http://www.kijkwijzer.nl/upload/download_pc/24.pdf |
| Brazil | Classificação Indicativa | https://www.justica.gov.br/seus-direitos/classificacao/guia-pratico/practical-guide.pdf |
| Bulgaria | Film Industry Act of 2003 administered by the National Film Rating Committee | https://web.archive.org/web/20150924012846/  http://www.filmmakersbg.org/zakon-kino-eng.htm |
| Canada | Culture et communications Quebec | https://mcc.gouv.qc.ca/index.php?id=5938 |
| Canada | Canadian motion picture rating system (MPA) | https://www.mpa-canada.org/film-ratings/ |
| Denmark | Media Council for Children and Young People | https://web.archive.org/web/20170104234653/http://www.dfi.dk/Service/English/Children-og-Youth/The-Media-Council-for-Children-and-Young-People-in-Denmark/Film-Classification/Film-Classification-System-and-Criteria.aspx |
| Finland | Finnish Centre for Media Education and Audiovisual Media | https://kavi.fi/en/printable-symbols-for-age-ratings/; http://www.meku.fi/ |
| France | Ministry of culture | https://www.cnc.fr/web/en/about/regulatory-function/film-classification |
| Germany | Freiwillige Selbstkontrolle der Filmwirtschaft  (FSK) | https://www.spio-fsk.de/?seitid=1287&tid=480 |
| Germany | Unterhaltungssoftware Selbstkontrolle (USK) | https://usk.de/en/home/age-classification-for-games-and-apps/games-and-apps-in-the-iarc-system/ |
| Hong Kong | Film Censorship Authority (FCA) | https://www.ofnaa.gov.hk/eng/aboutus/aboutus4b_2.htm |
| Iceland | FRÍSK (Félag rétthafa í sjónvarps- og kvikmyndaiðnaði) | https://translate.google.com/translate?hl=en&sl=is&u=http://frisk.klapptre.is/&prev=search&pto=aue |
| India | India Film Censor Board | https://www.cbfcindia.gov.in/main/certification.html |
| Indonesia | The Indonesian Game Rating System (IGRS) | https://igrs.id/tentang |
| Ireland | Irish Film Classification Office | http://www.ifco.ie/en/ifco/pages/film-12a |
| Jamaica | Cinematograph Act of 1913 | https://moj.gov.jm/laws/cinematograph-act |
| Japan | Computer Entertainment Rating Organisation (CERO) | https://www.cero.gr.jp/en/publics/index/17/ |
| Malaysia | Film Censorship Board | http://www.moha.gov.my/index.php/en/definisi/122-maklumat-korporat/maklumat-bahagian/lembaga-penapis-filem/188-film-classification |
| Maldives | National Bureau of Classification | https://web.archive.org/web/20140220024616/http://www.nbc.gov.mv/services/classification-ratings |
| Netherlands | Netherlands Institute for the Classification of Audiovisual Media (NICAM) | https://www.kijkwijzer.nl/kijkwijzer |
| New Zealand | Office of film and Literature Classification (OLFC) | https://www.classificationoffice.govt.nz/find-ratings/new-zealands-classification-labels/ |
| Nigeria | National Film and Video Censors Board | https://www.nfvcb.gov.ng/classification-symbols/; https://web.archive.org/web/20120224003112/http://nfvcb.gov.ng/pages.asp?pageid=352 |
| Norway | The Norwegian Media Authority (Medietilsynet) | https://medietilsynet.no/globalassets/aldersgrense-ikoner-og-veiledning-no-og-eng/200423-retningslinjer_aldersklassifisering_engelsk_pages.pdf |
| Malta | Film Board in accordance with the Malta Council for Culture and the Arts Act | http://justiceservices.gov.mt/DownloadDocument.aspx?app=lp&itemid=24242&l=1 |
| Philippines | Movie and Television Review and Classification Board | https://midas.mtrcb.gov.ph/ |
| Poland | Krajowa Rada Radiofonii i Telewizji (KRRiT) | http://www.krrit.gov.pl/en/for-viewers-and-radio-audience/minors-protection/ |
| Saudi Arabia | General Commission for Audiovisual Media (GCAM) | https://www.gcam.gov.sa/en/PoliciesAndGuidelines/Pages/ContentClassifications.aspx |
| Singapore | Infocomm Media Development Authority (IMDA) | https://www.imda.gov.sg/-/media/Imda/Files/Regulations-and-Licensing/Regulations/Codes-of-Practice/Codes-of-Practice-Media/Film-Classification-Guidelines-29_Apr_2019.pdf?la=en |
| South Africa | Film and Publication Board (FPB) | https://www.fpb.org.za/ratings/ |
| South Korea | The Korea Media Rating Board (KMRB) - Film and Video | http://www.kmrb.or.kr/eng/CMS/Contents/Contents.do?mCode=MN024 |
| South Korea | Game Rating and Administration Committee (GARC) | https://www.grac.or.kr/english/ |
| Sweden | Swedish Media Council (SMC) | https://www.statensmedierad.se/ovrigt/inenglish/filmclassification.580.html |
| Taiwan | Game Software Rating Management Regulations | https://law.moj.gov.tw/ENG/LawClass/LawAll.aspx?pcode=J0030086 |
| Turkey | Radio and Television Supreme Council | <https://www.rtukisaretler.gov.tr/AIsaretlerPublic/content?id=2&mid=4> |
| United Arab Emirates | National Media Council (NMC) | http://nmc.gov.ae/en-us/Media-Center/Events/Pages/Age-Classification-System.aspx |
| United Kingdom | British Board of Film Classification (BBFC) | https://www.bbfc.co.uk/sites/default/files/attachments/BBFC-Classification-Guidelines_0.pdf |
| United Kingdom | British Broadcasting Corporation (BBC) | http://downloads.bbc.co.uk/commissioning/site/Guidance_labels_final.pdf |
| United Kingdom | Ofcom | https://www.ofcom.org.uk/tv-radio-and-on-demand/broadcast-codes/broadcast-code/section-two-harm-offence |
| United Kingdom | Old Vic | https://www.oldvictheatre.com/your-visit/booking-tickets/trigger-warnings |
| United Kingdom | British Phonographic Industry (BPI) | https://www.bpi.co.uk/media/1047/parental-advisory-guidelines.pdf |
| United States of America | Motion Picture Association of America (MPAA) | https://www.motionpictures.org/film-ratings |
| United States of America | TV parental guidelines monitoring board | http://www.tvguidelines.org/resources/TV_Parental_Guidelines_Brochure.pdf |
| United States of America | National Public Radio (NPR) | https://www.npr.org/sections/therecord/2010/10/29/130905176/you-ask-we-answer-parental-advisory---why-when-how |
| INTERNATIONAL | Amazon | https://www.amazon.com/gp/help/customer/display.html?nodeId=G2C2CPZWGZWHZ42J |
| INTERNATIONAL | Apple Store | https://help.apple.com/app-store-connect/#/dev269f11291 |
| INTERNATIONAL | Blackberry | https://helpblog.blackberry.com/en/2012/09/app-world-content-ratings |
| INTERNATIONAL  Antigua and Barbuda, Argentina, Bahamas, Barbados, Belize, Bolivia, Canada, Chile, Colombia, Costa Rica, Cuba, Dominica, Dominican Republic, Ecuador, El Salvador, Greenland, Grenada, Guatemala, Guyana, Haiti, Honduras, Jamaica, Mexico, Nicaragua, Panama, Paraguay, Peru, Saint Kitts and Nevis, Saint Lucia, Saint Vincent and the Grenadines, Suriname, Trinidad and Tobago, United States of America, Uruguay and Venezuela) | Entertainment Software Rating Board (ESRB) | https://www.esrb.org/ratings-guide/ |
| INTERNATIONAL | Facebook | https://www.facebook.com/communitystandards/graphic_violence |
| INTERNATIONAL | Google Play | https://support.google.com/googleplay/answer/6209544 |
| INTERNATIONAL  Australian Classification Board (ACB), Classificação Indicativa (ClassInd) - Brazil, Entertainment Software Rating Board (ESRB) - North America, Game Rating and Administration Committee (GRAC) - South Korea, Pan European Game Information (PEGI) - Europe, Unterhaltungssoftware Selbstkontrolle (USK) – Germany | International Age Rating Coalition (IARC) | https://www.globalratings.com/ |
| INTERNATIONAL | Netflix | https://help.netflix.com/en/node/2064/bj |
| INTERNATIONAL  Austria, Belgium, Bulgaria, Cyprus, Czech Republic, Denmark, Estonia, Finland, France, Greece, Hungary, Iceland, Ireland, Israel, Italy, Latvia, Lithuania, Luxembourg, Malta, Netherlands, Norway, Poland, Portugal, Romania, Slovak Republic, Slovenia, Spain, Sweden, Switzerland and United Kingdom) | Pan European Game Information (PEGI) | https://pegi.info/what-do-the-labels-mean |
| INTERNATIONAL | Reddit | https://en.wikipedia.org/wiki/Not_safe_for_work https://www.reddithelp.com/en/categories/reddit-101/reddit-basics/what-do-all-these-acronyms-mean |
| INTERNATIONAL | Twitter | https://help.twitter.com/en/rules-and-policies/media-policy |
| INTERNATIONAL | YouTube | https://support.google.com/youtube/answer/146399?hl=en  https://support.google.com/youtube/answer/2802167?hl=en |
