# Supplementary material for: Typology of content warnings and trigger warnings: Systematic review
Source: PLoS One. 2022 May 4;17(5):e0266722. doi: 10.1371/journal.pone.0266722 (PMC9067675; doi:10.1371/journal.pone.0266722)
Supplement: S3 Appendix — (DOCX) [file pone.0266722.s004.docx]

**S3 Appendix**

**NEON content warning typology (complete)**

| **Warning** | **n** | **Definition of warning** | **Example warnings**  Text [reference in S2 Appendix Data Abstraction Table] |
| --- | --- | --- | --- |
| **CATEGORY 1: Violence** (n=536)  Definition: content contains violence | | | |
| 1.1 Violence | 91 | Warnings referring to general depictions of violence | *18SG - Contains elements of violence or horror, but not too excessive* [64] |
| 1.2 War | 35 | Warnings referring to depictions of warfare and combat | *Graphic depictions of war time violence, trauma* [131]  *Some forms of violence, including combat and bombings* [101] |
| 1.3 Weapons | 9 | Warnings referring to the use of/violence with guns, knives and other easily accessible weapons | *(Symbol) References to scenes involving aggressive conflict, may contain blood scenes(realistic blood, gore, weapons, and depictions of human injury and death)* [77] |
| 1.4 Terrorism | 4 | Warnings referring to terrorism or terrorist acts | *15: may contain realistic, authentic or intense depictions or scenes of war, catastrophes, or terrorism acts* [69] |
| 1.5 Police brutality | 2 | Warnings referring to police brutality | *Colonialism, racism, and police brutality* [62]  *The circulation of images of Black people’s deaths at the hands of police* [100] |
| 1.6 Motiveless killing | 10 | Warnings referring to motiveless killing (n=7) and violence towards defenceless people (n=3) | *PEGI Rating 18: Extreme violence -Multiple, motiveless killing -Violence towards defenceless people* [63] |
| 1.7 Sexual violence | 133 | Warnings referring to sexual violence including rape and sexual assault (n=105), gendered violence (n=2), violence in relationships (n=20), sexual threat and harassment (n=6), and sex trafficking (n=1) | *Sexual Violence: The degradation of an individual in a sexual manner. May contain images of non-consensual acts with the intent to inflict harm, for example, simulated rape, and/or the use of threat to force compliance in sexual activity* [89] |
| 1.8 Animal cruelty | 7 | Warnings referring to violence towards animals including animal torture and abuse (n=3), killing (n=2), or suffering (n=2) | *Imagery of animals in a ritual slaughter context showing dismemberment, or visible innards, or charring or burning* [49] |
| 1.9 Torture | 26 | Warnings referring to content that shows the infliction of pain or injury including serious human injury (n=17), infliction of pain (n=3), assault (n=3), and mutilation (n=3) | *Imagery of non-medical foreign objects (such as metal objects, knives, nails) involuntarily inserted or stuck into people causing grievous injury* [49] |
| 1.10 Genocide | 4 | Content referring to genocide and the deliberate killing of a large group of people, especially those of a particular ethnic, religious, or national grouping | *The role of war in colonialism and genocide*…*Ethnic cleansing and genocide…*[101] |
| **CATEGORY 2: Sex** (n=332)  Definition: content contains sexual themes, including nudity, sexual content and relationships | | | |
| 2.1 Nudity | 134 | Warnings referring to nudity including partial, side-profile, upper-body, full-frontal, prolonged, sexualised/erotic. As well as reference to non-sexualised nudity and nudity which is educational or medical in nature | *Nudity – The film may contain occasional, as well as natural non-sexual nudity* [71]  *Sexual Content - Non-explicit depictions of sexual behavior, possibly including partial nudity. Strong Sexual Content - Explicit and/or frequent depictions of sexual behavior, possibly including nudity* [63] |
| 2.2 Mild sexual content | 12 | Warnings referring to implied or mild sexual activity, including kissing or hugging, sexual posturing, and sexual innuendos | *Sex: G - Brief displays of affection (e.g. brief kissing). PG - Displays of affection (e.g. kissing and caressing). Brief, infrequent and discreet references to sexual activity* [74]  *Contains expressions of romance or love. (Possibly includes kissing, hugging, dating, and other expressions of romantic desire or relations* [53] |
| 2.3 Explicit sexual content | 198 | Warnings referring to explicit sexual themes or content including sexual scenes and activities | *'Adults Only’ (AO): Games for adult users that include prolonged scenes of intense violence and/or graphic sexual content* [21]  *M – MATURE: appropriate for readers age 17 and older. May contain intense violence, extensive profanity, nudity, sexual themes and other content suitable only for older readers* [59] |
| 2.4 Relationship conflict | 8 | Warnings referring to conflict and issues in relationships including adultery (n=2), unsafe/unstable family relations (n=1), social rejection (n=1), strong emotional outbursts in relationship contexts (n=2), and sexual vulnerability (n=1) | *Age 12: may contain some unsettling themes related to the “adult realm”, such as depictions or portrayals of relationship conflicts, sexual acts or strong emotional outbursts* [69] |
| 2.5 Reproductive health | 14 | Warnings referring to content that displays situations or scenes associated with reproductive health including abortion (n=7), child birth (n=3), pregnancy (n=2), contraception (n=1), miscarriage, and foetuses (n=1) | *Graphic imagery and discussions of sexual assault and sexual abuse, violence, and/or abortion and contraception* [7] |
| **WARNING CATEGORY 3: Stigma** (n=328)  Definition: content depicts negative stereotypes about or attitudes towards a specific group, such as racism or sexism | | | |
| 3.1 Racism | 66 | Prejudice, antagonism or discrimination towards a person or group of people of a particular racial or ethnic group. Warnings include, racist slurs (n=45), racial tension (n=6), race-based discrimination (n=3), racial violence (n=12) and hate crimes (n=1) | *Racism, US imperialism, sexism* [113]  *Warns of scenes or language that is biased or prejudiced with regard to race, ethnicity, gender, religion, sexual orientation or other identifiable group characteristics* [75] |
| 3.2 Anti-religious | 20 | Prejudice, antagonism or discrimination towards a person or group of people of a particular religion including religious stereotypes (n=19) | *Discrimination: the game contains depictions of ethnic, religious, nationalistic, or other stereotypes likely to encourage hatred* [41] |
| 3.2.1 Anti-Semitic | 3 | Prejudice, antagonism or discrimination towards Judaism and/or people identifying as Jewish either as a religious or racial group | *Content on racism, anti-Semitism, sexual assault* [101] |
| 3.2.2 Anti-Christian | 1 | Prejudice, antagonism or discrimination towards Christianity and/or people identifying as Christians | *Blood, Christian demonology, and body horror* [30] |
| 3.2.3 Islamophobia | 1 | Prejudice, antagonism or discrimination towards Islam and/or people identifying as Muslims | *Anti-Muslim / Islamophobic language* [62] |
| 3.3 Colonialism | 25 | Content referring to acts of colonialism including colonial and police brutality, violent fantasies, and oppression | *Religious persecution and colonial oppression* [15]  *Colonialism, racism, and police brutality* [62] |
| 3.3.1 Slavery | 4 | Content referring to acts of slavery including the history of slavery and modern-day slavery | *Abuse, racism (especially the history of slavery in the US)* [30]  *Slavery, reconstruction and the civil rights movement, graphic photos of lynching victims* [107] |
| 3.4 Classism | 27 | Prejudice, antagonism or discrimination towards people belonging to a particular social class including classism (n=22) and privilege and oppression (n=10) | *Oppressive structures such as racism, sexism, colonialism, classism* [92]  *Classism, sexism, heterosexism, cissexism, and ableism [15]* |
| 3.5 Sexism | 43 | Prejudice, antagonism or discrimination on the basis of sex and/or gender | *Hate crimes, sexism, sexism culture* [126]  *Sexism, racism, violence* [20] |
| 3.5.1 Misogyny | 15 | Prejudice, antagonism or discrimination directed towards females | *Violent misogyny, and reproducing that misogynistic violence* [100]  *Troubling content, particularly on rape and sexual violence (but also other forms of physical and social trauma linked to misogyny and racism)* [101]  *Gory, abusive and misogynistic violence* [15] |
| 3.5.2 Misandry | 1 | Prejudice, antagonism or discrimination directed towards males | *Cis-sexism, sexism, misandry, and misogyny [14]* |
| 3.6 Transphobia | 23 | Prejudice, antagonism or discrimination against people identifying as or perceived to be transsexual or transgender including cis-sexism (n=13) | *Transphobia and trans misogyny* [30]  *Cissexism, transphobic violence* [62] |
| 3.7 Gender-identity | 4 | Warnings referring to content that depicts gender-identity issues including gender dysphoria | *Violence, sex, gender identity, and race* [110]  *Gender dysphoria, depression / suicidal ideas* [119] |
| 3.8 Sexuality | 30 | Warnings referring to content that depicts sexuality issues | *Gender, race, and sexuality* [100]  *Violence, sexuality and abusive language* [95]  *Race, class, citizenship, gender and sexuality* [5] |
| 3.8.1 Homophobia | 53 | Prejudice, antagonism or discrimination towards people identifying or perceived to be LGBTQ+ including heterosexism (n=14) | *Discrimination: The work as a whole must not endorse discriminatory language or behaviour, although there may be racist, homophobic or other discriminatory themes and language* [79]  *Child abuse, LGBTQ discrimination* [22] |
| 3.9 Anti-disability | 16 | Prejudice, antagonism and discrimination based on disability including ableism (n=13) and neuro-atypical or neurodiverse shaming (n=1) | *Race, sexual orientation, disability, colonialism, torture* [13]  *Discussion of ‘isms,’ neuroatypical shaming, slurs* [1] |
| **CATEGORY 4: Disturbing Content** (n=236)  Definition: content contains imagery, sounds, or effects that may frighten, disgust or scare | | | |
| 4.1 Disturbing content with threat | 26 | Disturbing imagery, sounds or effects that contain threat or threatening situations (n=26) | *Some dark, dramatic or moderately threatening scenes, scenes with dramatic or powerful effects (sound or images), brief scary elements (characters or situations)* [69] |
| 4.2 Horror and terror | 90 | Imagery, sounds or effects that may frighten or scare including horror and terror themes or situations | *Scenes in the film consists elements of horror; scary; negative acts; suspense and frantic elements, but not excessive; elusive storyline; and elements that can disturb a child’s emotion* [64] |
| 4.3 Disturbing imagery | 108 | Content that shows or describes bloody and gory imagery including dismemberment (n=7), severe wounds (n=6), bloodletting (n=4), visible innards (n=3), and cannibalism (n=2) | *For the following content, we include a warning screen so that people are aware that the content may be disturbing… Dismemberment,*  *Visible internal organs; partially decomposed bodies,*  *Charred or burning people,*  *Victims of cannibalism, Throat-slitting* [49] |
| 4.4 Medical content | 9 | Content that contains medical footage, including treatment (n=5), graphic medical scenes (n=2), medical foreign objects (n=1), and dental trauma (n=1) | *Infrequent or mild medical or treatment-focused content* [58]  *Similarly, mildly violent or disturbing content may contain a small amount of other imagery or situations that are disturbing or repulsive to sensitive viewers. Examples include real or dramatized medical footage, or depictions of disgusting or scary content in a horror or fantasy context* [50] |
| 4.5 Human bodies and functions | 3 | Content that contains human bodies and bodily functions including vomit (n=2), scarification (n=1) and defecation | *Vomit, warfare and weapons* [129]  *Childbirth, blood, scarification* [1] |
| **CATEGORY 5: Language** (n=235)  Definition: content contains language which is sexual, crude or offensive | | | |
| 5.1 Sexual language | 82 | Warnings referring to the use of sexual language or gestures including innuendo and sexual imagery | *Infrequent and non-detailed use of sexual humour or sexual innuendoes, including crude hand gestures and sexual imagery* [73]  *This content descriptor can accompany a PEGI 12 rating if the game includes sexual posturing or innuendo* [41] |
| 5.2 Adult humour | 40 | Warnings referring to the use of adult humour | *LA - contains adult humour* [80]  *Suggestive themes, Alcohol, Mature humour* [63] |
| 5.3 Swearing | 38 | Warnings referring to the use of expletives and swear words | *The use of strong swear words or sexually-derived or suggestive expletives and expressions as well as the use of swear words and expletives shall be allowed; provided that its use is infrequent and not vulgar* [71]  *Language – The film may contain dialogue or other word representations beyond polite language, but profane, offensive, and sexually-suggestive language or gestures shall not be allowed [71]* |
| 5.4 Offensive language | 75 | Warnings referring to the use of offensive language, including abusive (n=3), derogatory (n=3), sacrilegious (n=2) and controversial language (n=2) | *Language: contains profane, derogatory or bigoted language* [53]  *he M means the movie might contain violence, offensive language, drug use, sexual or adult themes or nudity* [67]  *Contains language that may be offensive to some groups, (i.e. sacrilegious language such as Goddamn)* [89] |
| **CATEGORY 6: Risky Behaviours** (n=200)  Definition: content depicts risky lifestyle behaviours | | | |
| 6.1 Drug misuse | 131 | Warnings referring to drug misuse, including illegal/prohibited drug use and abuse including drug addiction (n=1) | *Mild drug use: Contains mild drug use, including excessive or persistent consumption of alcohol or tobacco. It also may include incidental or comedic use of drugs such as marijuana, sativa, hallucinogens, or prescription pharmaceuticals, and implied, non-graphic use of other drugs, such as heroin* [50] |
| 6.2 Alcohol misuse | 28 | Warnings referring to alcohol misuse and consumption, including drunk driving (n=1) and alcohol dependency (n=1) | *(Symbol) - Alcohol, tobacco, drug: Reference to images of alcoholic beverages, tobacco products, illegal drug use* [77] |
| 6.3 Tobacco | 21 | Warnings referring to images, consumption or use of tobacco or cigars | *Drinking & Smoking: contains depiction or references to the consumption of alcohol and/or cigarette or cigar smoking* [53]  *Frequent or intense references to alcohol, tobacco, or drug use* [58] |
| 6.4 Gambling | 20 | Warnings referring to gambling including simulated gambling, betting or contests | *PEGI Gambling: the game contains elements that encourage or teach gambling. These simulations of gambling refer to games of chance that are normally carried out in casinos or gambling halls* [41]  *(Symbol) - gambling (betting): References to gambling or betting (speculation), simulated gambling* [77] |
| **WARNING CATEGORY 7: Mental Health** (n=108)  Definition: content relates to mental health issues | | | |
| 7.1 Mental health | 108 | Warning referring to mental health problems including content showing people experiencing mental health problems | *Experiences of psychosocial disability/mental illness, and discussion, but no graphic description of suicide* [33]  *Mental health, sexual violence* [116] |
| 7.2 Eating disorders | 18 | Warnings referring to eating disorders or disordered eating including body shaming (n=2), fat phobia/fat hatred (n=2), calories in food items (n=2) and references to weight (n=1) | *Eating disordered behavior or body shaming* [98]  *Extended accounts of abuse, disordered eating, self-harm* [113] |
| 7.3 Trauma | 15 | Warnings referring to trauma or traumatic issues including Post-traumatic stress disorder (PTSD) (n=3) and language of trauma (n=1) | *Veteran’s experiences of trauma* [22]  *Trigger warnings could provide necessary consideration for students living with everyday trauma, including survivors of endemic racialized, classed, and gendered violence* [100] |
| 7.4 Self-harm and suicide | 55 | Warnings referring to suicidal thoughts and ideas, and self-harm or self-injurious behaviours | *Dangerous behaviour (for example, suicide, self-harming and asphyxiation)* [80]  *NC16 - Mature themes that are appropriate for viewers aged 16 years and above. Themes such as self-harm or euthanasia should be treated with discretion* [74] |
| 7.5 Depression | 3 | Warnings referring to depression | *Mental torture - depression, potential for triggering panic as harm, eating disorders* [123]  *Sexual assault, depression, suicide* [117] |
| 7.6 OCD | 1 | Warnings referring to obsessive compulsive disorder (OCD) | *"Anything that might inspire intrusive thoughts in people with OCD”* [14] |
| 7.7 Panic attacks | 1 | Warnings referring to panic attacks | *Mental torture - depression, potential for triggering panic as harm* [123] |
| 7.8 Anxiety | 12 | Warnings referring to anxiety and phobias |  |
| 7.8.1 Spiders | 3 | Warning related to fear of spiders (arachnophobia) | *…death or dying, spiders…*[1] |
| 7.8.2 Snakes | 2 | Warning related to fear of snakes (ophidiophobia) | *…pregnancy, slimy things, snakes…*[129] |
| 7.8.3 Insects | 2 | Warning related to fear of insects (entomophobia) | *…food, gore, insects…*[129] |
| 7.8.4 Needles | 2 | Warning related to fear of needles or injections (trypanophobia) | *…skulls, skeletons, needles…* [1] |
| 7.8.5 Eye contact | 1 | Warning related to fear of eye contact/ being stared at (scopophobia) | *…drug use or talk of drugs (legal, illegal of psychiatric) eye contact ( Scopophobia…* [129] |
| 7.8.6 Irregular patterns | 2 | Warning related to fear of irregular patterns or clusters of small holes or bumps (trypophobia) | *...trypophobia (fear or disgust of closely-packed holes e.g. looking at head of lotus seed pod or strawberry)* [129] |
| 7.9 Hair pulling | 1 | Warning related to the impulse control disorder of hair pulling (trichotillomania) | *…spiders, trichotillomania (hair pulling)…* [129] |
| **CATEGORY 8: Death** (n=49)  Definition: content relates to human death or dying | | | |
| 8.1 Death | 41 | Warnings referring to content that depicts human death, including euthanasia (n=2), the death penalty and dying (n=25), dead bodies (n=6), corpses (n=4), capital punishment and executions (n=2), and skulls and skeletons (n=2) | *Photographs of dead bodies, extended accounts of abuse* [113]  *Violence, including the death penalty* [101] |
| 8.2 Accidents | 7 | Warnings referring to content that depicts fatal accidents leading to death or injury, including car and plane crashes (n=2) | *A fatal car crash/violent domestic abuse* [105]  *Videos that show the violent death of a person or people by accident or murder* [49] |
| 8.3 Natural disasters | 5 | Warnings referring to content that depicts natural disasters, including earthquakes, volcano eruptions and life-threatening weather conditions | *War, crime, gangs, science fiction, natural disasters* [73] |
| **CATEGORY 9: Parental Guidance** (n=47)  Definition: content may not be appropriate for children | | | |
| 9.1 Online access | 8 | Warnings that the software allows online interactions with other people and access to content that may be inappropriate | *PEGI Online: may contain online interactions* [41] |
| 9.2 Cyber-bullying | 2 | Warnings referring to content that includes reference to cyber-bullying and online harassment | *Online harassment, queerphobia, and sexual assault* [31] |
| 9.3 Competitive content | 1 | Warnings to indicate the content is intense and competitive with various game levels in order to gain rewards and incentives | *CI – Competitive Intensity, is the degree to which a player gets personally involved, and the level of excitement created in the players as they engage with the various game levels in order to gain incentives and rewards* [75] |
| 9.4 Imitative content | 4 | Warnings to signpost parents that content contains behaviours or acts that could be imitated by children | *IAT – Imitative Acts or Techniques, where there are dangerous act or techniques that may be copied or imitate, especially by children* [75] |
| 9.5 Upsetting content | 10 | Warnings referring to content that may upset viewers, including sad or sensitive themes | *May contain content that children find confusing or upsetting and may require the guidance of parents, teachers or guardians* [35]  *PG - Parental guidance suggested: Some material may not be suitable for children. Parents urged to give "parental guidance." May contain some material parents might not like for their young children* [83] |
| 9.6 Non-realistic violence | 30 | Warnings referring to non-realistic forms of violence, including slapstick (n=1), fantasy violence (n=29) and comic mischief (n=4) | *PEGI 7 this can only be non-realistic or non-detailed violence. Games rated PEGI 12 can include violence in a fantasy environment or non-realistic violence towards human-like characters* [41]  *E – EVERYONE: Appropriate for readers of all ages. May contain cartoon violence and/or some comic mischief* [59] |
| **CATEGORY 10: Crime** (n=38)  Definition: content depicts or relates to criminal activity | | | |
| 10.1 Crime | 38 | Warnings for content that includes criminal activity or dangerous criminal behaviours including criminal acts (n=17), dangerous or anti-social behaviour (n=16), kidnapping (n=3), gangs (n=1), murder (n=3) and trafficking (n=1) | *Crime: contains criminal activity, either by depiction or in interactive form. (Possibly includes illegal activity, dangerous and unlawful behavior, abusive behavior, prostitution, rape, organized crime, and other criminal acts.)* [53] |
| **CATEGORY 11: Abuse** (n=37)  Definition: content depicts or relates to abuse | | | |
| 11.1 Child abuse | 17 | Warnings referring to child abuse, including sexual/physical abuse (n=15), children suffering or in difficulty e.g. bullying (n=1), and paedophilia (n=1) | *PG13 - Themes should be suitable for teenagers aged 13 years and above. Dark themes such as horror or child abuse should be treated with discretion* [74]  *Scenes featuring explicit sex, incest, paedophilia, praising of the use of illegal drugs and violence of a strong imagery impact* [37] |
| 11.2 Emotional abuse | 5 | Warnings referring to emotional or mental abuse, including psychological violence (n=2), emotional suffering (n=1), mental abuse (n=1), victim blaming (n=1), and verbal abuse (n=1) | *Where lyrics or content of a recording or music video commercially released contains:*  *strong or coarse language, depictions of, or reference to, violence or physical or mental abuse* [82] |
| 11.3 Physical/sexual abuse | 7 | Warnings referring to physical and sexual abuse, including sexual misconduct (n=2), sexual coercion (n=1), and degradation in a sexual manner (n=1) | *Material that concerns sexual abuse or sexual assault, that is potentially racially or politically offensive, or graphically violent or sexual* [97]  *Abuse (e.g., physical, sexual, verbal, emotional)* [7] |
| 11.4 Neglect | 4 | Warnings referring to deliberate or unintentional neglect or abandonment (n=2) | *Other themes such as intense/ realistic depictions of war, catastrophes, terrorism, gross neglect, assault, suicide* [69] |
| **CATEGORY 12: Sociopolitical** (n=27)  Definition: content includes social and political issues | | | |
| 12.1 Injustice | 10 | Warnings referring to societal issues including poverty, justice, equality, justice, ethics, perversions of power, marginality, identity, citizenship, and discussion of inequalities | *Messages about identity, religion, science, ethics, justice, equality, and other topics that contemporary people find naive or offensive* [13] |
| 12.2 Political issues | 11 | Warnings referring to political issues, aspects and opinions including anti-governmental messages (n=4) and capitalism (n=2) | *(Symbol) - Crime, anti-societal or anti-governmental messages: Reference to images of crime, anti-societal or anti-governmental messages* [77]  *The film contains…social, sensitive political and religious elements which require a high level understanding* [64] |
| 12.3 Nazism | 2 | Warnings referring to content that contains discussions/support of Nazism including Nazi objects or clothing | *Blood, scarification, Nazi paraphernalia, slimy things [and] holes* [1] |
| 12.4 Class issues | 4 | Warnings referring to issues relating to class | *Sex, race, class, capitalism, and colonialism* [62] |
| **CATEGORY 13: Flashing Lights** (n=27)  Definition: content includes strobe or flashing lighting | | | |
| 13.1 Flashing lights | 12 | Warnings indicating the following content displays flashing lights | *Flashing or strobing light: Contains flashing light or other strobing content that could affect individuals with photosensitive epilepsy* [50] |
| **CATEGORY 14: Objects** (n=4)  Definition: content contains specific objects | | | |
| 14.1 Objects | 2 | Warnings referring to content that contains specific objects including slimy objects (n=2), indigenous artefacts (n=1), and drones (n=1). | *…Nazi paraphernalia, slimy things [and] holes* [1]  *…Suicide in a ballet, indigenous artefacts, images of dead bodies… [13]*  *…Stand Your Ground laws, drones, homophobia … corpses, skulls, skeletons…[1]* |
